# EPIGENETIC PROFILES OF BIOLOGICAL AGING HALLMARKS

**DOI:** 10.1093/geroni/igz038.1584

**Published:** 2019-11-08

**Authors:** Diana L Leung, Zuyun Liu, Morgan E Levine

**Affiliations:** 1 Yale University, New Haven, Connecticut, United States; 2 Department of Pathology, Yale School of Medicine, New Haven, Connecticut, United States; 3 Yale University School of Medicine, New Haven, Connecticut, United States

## Abstract

Investigation into the hallmarks of aging point to the existence of shared mechanisms that underlie the biological aging process. While there is a general consensus that hallmarks of aging rarely occur in isolation, little is known in regards to their overlapping networks or how interactions contribute to manifestations at the clinical level. Here, we examine whether shared epigenetic alterations—one of the proposed hallmark of aging—underlies diverse conditions characterized by other hallmarks, including cellular senescence, loss of proteostasis, genomic instability, mitochondrial dysfunction, and inflammation. Using weighted network analysis, we identified consistent overlaps in the methylation profiles across the different traits. For instance, epigenetic modules that were distinct in senescence were also affected in progeroid syndromes (Hutchinson-Gilford Progeria Syndrome and Werner’s Syndrome) and smokers. These CpGs tended to be located in CpG islands, which are notable for their strong association with transcriptional regulation. Overall, our results suggest that epigenetic alterations intersect with various hallmarks of aging. In moving forward, incorporation of this understanding may lead to the development of biomarkers that better capture the biological (rather than chronological) aging process.

